# Noninvasive Evaluation of Portal Hypertension Using a Supervised Learning Technique

**DOI:** 10.1155/2017/6183714

**Published:** 2017-10-12

**Authors:** Mindaugas Marozas, Romanas Zykus, Andrius Sakalauskas, Limas Kupčinskas, Arūnas Lukoševičius

**Affiliations:** ^1^Biomedical Engineering Institute, Kaunas University of Technology, Kaunas, Lithuania; ^2^Gastroenterology Department, Lithuanian University of Health Sciences, Kaunas, Lithuania

## Abstract

Portal hypertension (PHT) is a key event in the evolution of different chronic liver diseases and leads to the morbidity and mortality of patients. The traditional reliable PHT evaluation method is a hepatic venous pressure gradient (HVPG) measurement, which is invasive and not always available or acceptable to patients. The HVPG measurement is relatively expensive and depends on the experience of the physician. There are many potential noninvasive methods to predict PHT, of which liver transient elastography is determined to be the most accurate; however, even transient elastography lacks the accuracy to be a perfect noninvasive diagnostic method of PHT. In this research, we are focusing on noninvasive PHT assessment methods that rely on selected best-supervised learning algorithms which use a wide set of noninvasively obtained data, including demographical, clinical, laboratory, instrumental, and transient elastography measurements. In order to build the best performing classification meta-algorithm, a set of 21 classification algorithms have been tested. The problem was expanded by selecting the best performing clinical attributes using algorithm-specific filtering methods that give the lowest error rate to predict clinically significant PHT. The suggested meta-algorithm objectively outperforms other methods found in literature and can be a good substitute for invasive PHT evaluation methods.

## 1. Introduction

The standard way of evaluating PHT in patients with chronic liver disease is measuring HVPG [1]. Measurement of HVPG is an invasive and inconvenient procedure where the difference between wedged venous pressure and free haptic venous pressure is taken [2]. An HVPG value between 1 mmHg and 5 mmHg is considered normal. Higher pressure is defined as PHT [3, 4]. Different values of HVPG indicate different risks for complications in patients with chronic liver diseases [5]. The standard threshold value for development of complications is HVPG > 10 mmHg and is considered as clinically significant PHT. It is associated with the risk of esophageal varices formation, clinical decompensation, development of hepatocellular carcinoma (HCC), or death after liver resection due to it [6–9]. HVPG > 12 mmHg is considered to be severe PHT and is linked with a higher risk of acute variceal bleeding [10, 11]. The main problem lies in the fact that there are no reliable and noninvasively obtained biomarkers or methods that could be substituted for the invasive HVPG measurement with sufficient accuracy.

Available noninvasive methods of indirect evaluation of PHT involves several techniques, including clinical examination, abdominal ultrasound with or without Doppler investigation, CT and MRI scans, evaluation for oesophageal varices, and measuring liver and spleen stiffness using different techniques [12–15]. The most promising results are revealed in liver elastography in the form of transient elastography. Liver transient elastography demonstrated quite high sensitivity, specificity, and accuracy (0.88, 0.87, and 0.88, resp.) to predict clinically significant PHT [14], but not enough to replace the invasive HVPG measurement.

The ideal method for diagnosing PHT should be safe, quantitative, accurate, objective, reproducible, inexpensive, and validated. None of today's available methods fully meets these criteria; however, the combination of different methods using supervised learning algorithms and a wide set of noninvasively available data could overcome these limitations.

Several studies were devoted to finding a potential noninvasive method to classify patients, with and without PHT, using different learning algorithms, with contradictory results [16–19]. Disagreement between various studies could be explained by different models of analysis and different data inputs used to generate results.

In this paper, the focus is on the classification problem that involves noninvasively obtained clinical data. Development of an objective classification meta-algorithm would appear to be a reliable noninvasive method to classify patients with and without PHT and would open possibilities for an easily accessible, less exhausting examination that would be less expensive, safer, quantitative, and reproducible.

The goal of the research is to select and prepare a meta-algorithm for classifying patients into groups with different HVPG values using noninvasively obtained clinical data. The tasks include the following:

- Selecting the best classifying meta-algorithm by testing the most common classifiers with noninvasively obtained data
- Ranking/selecting an optimal number of attributes (data subsets) that perform best for the selected algorithm

## 2. Materials and Methods

### 2.1. Dataset

Data from the original study published in the paper by Zykus et al. [14] was used in this study. The primary goal of the original paper was to analyze the relationship between liver/spleen transient elastography and HVPG. All data used for this paper was collected prospectively as leading data to elastography results during the original study.

In this research, the dataset consists of records from 107 patients with chronical liver disease that were referred for HVPG measurement. Patients had clinical examinations and laboratory investigations leading to 24 attributes (Table 1) containing demographical, clinical, serum laboratory, spleen geometrical, transient elastography (TE), blood stream, and vein data acquired by abdominal ultrasound. Invasively measured HVPG was taken as a reference in our investigation. Bioethical Committee approval and patient consents were obtained before collecting clinical data.

The routine clinical (weight, height, and cause of chronic liver disease), hematological (complete blood count), and biochemical (bilirubin, albumin, prothrombin time, ALT, and AST) investigations were performed at the same day prior to HVPG measurement. Abdominal ultrasonography was performed to exclude multiple focal liver lesions, and various ultrasound-based parameters were recorded (presence of umbilical or pararenal shunt, portal vein width, portal blood flow mean peak velocity, portal blood flow mean velocity, portal blood flow type (hepatopetal versus hepatofugal), hepatic vein blood flow type (triphasic, biphasic, or monophasic), hepatic vein damping index, and splenic vein size (width, length, and thickness)).

Liver stiffness using FIBROSCAN® (Echosens, Paris, France) device was measured on the same day before HVPG measurement. Patients were in fasting state. Procedure was performed in accordance with manufacturer's recommendations. Interquartile range/median < 30% and success rate > 60% were considered as good-quality criteria for TE. We performed 10 successful measurements for each patient.

Assessment of spleen stiffness was performed by the same methodology used for liver elastography. The quality criterion (interquartile range/median, success rate, and number of successful measurements) for spleen stiffness was the same as for liver stiffness. If typical elastography picture could not be found using FIBROSCAN device, exact point for spleen stiffness measurement was found using Toshiba Xario 200 ultrasound device (Toshiba Medical Systems Corporation, Japan).

HVPG was measured in fasting state. None of the patients have received medications affecting portal pressure before HVPG measurement. HPVG was measured using catheter wedge technique by experienced radiologists using Judkins right 6 fr catheter (Boston Scientific, USA, Marlborough). Right hepatic vein was selectively cannulated and catheter position confirmed by vein angiogram. The occluded position of the catheter was checked by the absence of reflux after the injection of 2 mL of a contrast medium and appearance of sinusoidogram (Infinity R50, Drager, Germany). The mean of at least 3 readings was taken for further analysis. If the difference between the readings was greater than 1 mmHg, all the previous recordings were cancelled, and new readings were taken. Radiologist was blinded to clinical data and liver/spleen stiffness results.

39.25% of records in the collected clinical dataset have missing values. Missing data appears to be grouped in clusters for each patient record, for instance, in 6.54% of records, all demographic data is missing; in 12.15% of records, blood test results are missing; in 12% of records, Doppler test results are missing; in 25.30% of records, hepatic vein data is missing; and in 7.48% of records, there is a lack of spleen stiffness data.

### 2.2. Classification Aims, Criteria, and Classes

The dataset has been divided into two groups based on the measured HVPG value [1]. This decision is based on a gold-standard method which assesses clinically significant PHT (CSPH) exceeding an HVPG value of 10 mmHg [12]. CSPH is selected as the threshold for dividing patients in to two classes:

1. HVPG <10 mmHg—patients have no gastroesophageal varices and have low risk of developing them in next five years.
2. HVPG ≥10 mmHg—patients have high risk of severe complications starting with variceal bleeding, ascites, or clinical decompensation liver cirrhosis, ending with HCC.

The dataset contains 107 records and has some asymmetry between classes. Into class 1 falls 27% of records, and into class 2 falls 73% of records. The dataset is not considered highly imbalanced, but classification accuracy (Acc) should not be used as the main performance evaluation criteria. Instead, a more objective classification performance measurement parameter, area under the ROC curve (AUC), has been selected [20]. When searching similar work in literature, there are more metrics, such as sensitivity (Sn) and specificity (Sp), used to indicate performance of different methods. Those metrics are also included in this research for better comparison.

### 2.3. Coping with Missing Data

Most classifiers are capable of running on a dataset with missing data. In order to test a scenario with complete data, there is a need to filter out records with missing values. The most radical way of coping with missing data is to reject records that have missing data. The problem is that by doing so, we may lose some portion of records that might have some influence in classifier training accuracy.

In many cases, missing values are filled using statistical imputation methods like mean or distribution-based imputation. Distribution method appears to be more accurate and unbiased as compared to mean-based imputation method [21]. The more advanced missing value imputation methods use prediction models maximum-likelihood of possible missing values. However, there is a risk that predicting missing attributes by using similar information can be useless and even harmful taking into account specific and sensitive clinical data of the medical case. When the number of missing values in the dataset is raised, there is a risk to oversimplify the problem. If missing values in the dataset are not randomly distributed, there is a risk to create invalid knowledge [22, 23].

In order to test the impact of imputation of missing values, the K-Nearest Neighbor (KNN) method with Euclidian distance function was implemented. The algorithm selects the most common value among all neighbors.

**Figure 1:**
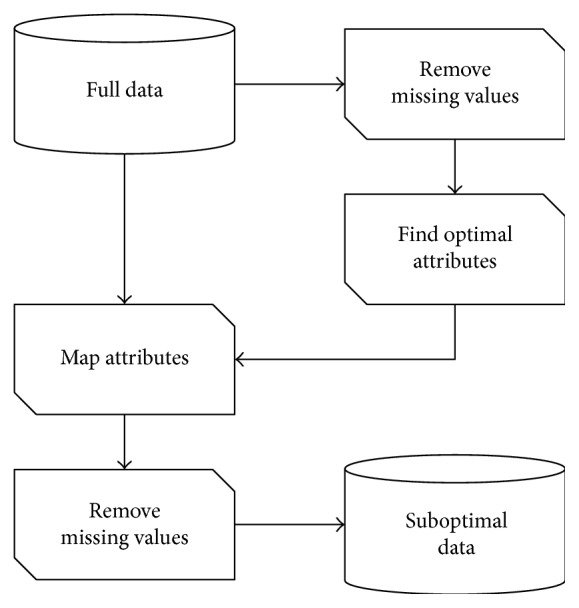
Selecting important attributes without missing data.

Since dataset is small, and most of missing values are clustered, in order to get the maximum number of data records without missing values, an algorithm was proposed, which works in two stages (Figure 1). First, missing data is removed from the full dataset, then optimal attributes are selected using a wrapper method. Next, an optimal attribute set is mapped into the full dataset and missing values are removed from only selected attributes. This way, we should end up with a suboptimal attribute set and a maximum number of records without missing values.

### 2.4. Optimal Feature Set Selection

The second problem that we were focusing on was finding the best performing attributes used in classification algorithms. This way, we could reduce the dimensionality of data that can cause redundancy and noise [24]. A reduced number of attributes can improve performance, increase accuracy, reduce overfitting, and, at the same time, reduce the cost of diagnosis. By reducing the attribute number, there is also a risk of lowering the accuracy of such algorithms as decision trees. Two attribute selection methods that are different in their nature have been used.

The first attribute selection method is relief filtering, which is a classifier-independent attribute ranker indicating the relevance of attributes to a target concept/threshold [25, 26]. The problem with relief filtering is that it may rank out valuable parameters that may have a significant influence in some particular classification algorithm. The relief filtering method could be useful when deciding which missing attributes (holes) in the dataset are more “influential.” The highest ranked missing attributes should be omitted from classification, while lower ranked attributes will most likely have lower influence and thus could be left for the classification task.

The second attribute selection method is known as wrapper and is classifier-dependent. This is an iterative attribute selection routine using a cross-validation method to estimate the accuracy of a classifier for a given set of attributes and, one by one, selects the best performing classifiers. It is possible to select the best attribute subset for each algorithm [27]. The attribute search is terminated with a user selectable threshold value which is a standard deviation of the mean across multiple cross-validation runs. The default threshold value in all tests is selected to be 1%. The wrapper-based attribute selection method usually leads to a differing set of optimal attributes for different classifiers. This may be an obstacle when implementing a meta-algorithm having multiple classifiers. To overcome this, a joined subset of attributes was used in the meta-algorithm.

### 2.5. Algorithms Used

Data mining software Weka, hosted by University of Waikato, has an extensive set of machine learning tools and methods that fit our needs and has been the tool of our choice [28]. After preliminary analysis, five groups of the most effective algorithms, including Naïve Bayes (NB), regression, nearest neighbor, rule based, and decision trees, have been selected (Table 2).

**Figure 2:**
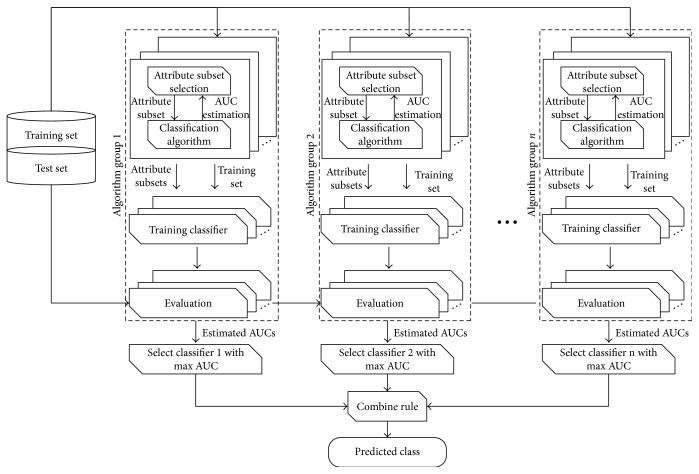
The meta-algorithm selects the best performing classifiers and optimal sets of attributes from predefined groups. Each best classifier is then trained and results are combined into a final prediction.

Each algorithm, on its own, has pros and cons [29–31]. An ensemble meta-algorithm seemed necessary, especially for small datasets. Using a single basic classifier, there is a risk of overfitting data or getting algorithm-specific errors. In the first stage of the meta-algorithm, the best performing classifier in each algorithm group, along with the best performing attributes, are selected by the highest AUC value. As seen in Figure 2, each selected algorithm of its group is trained consecutively using selected attributes and results that are then are combined in order to predict the final class. Each algorithm performs a 10-fold cross-validation in order to get a more generalized and independent dataset leading to less overfitting.

Five different combining rules, including averaging, product, majority voting, minimum probability, and maximum probability, have been included for finding the best output. Testing this algorithm should reveal if it could reduce classification error and give good classification results. The meta-algorithm can be expanded to have any number of algorithm groups, with any number of algorithms in each group. It is also not data or attribute number dependent. This detailed meta-algorithm could be taken as a pseudocode, which shows inherent flexibility and easy evolvement of the meta-algorithm with the emergence of new classifiers, as well as with new data, when available.

## 3. Results

In order to test algorithm performance, four scenarios have been implemented: I—data without missing values; II—unprocessed (full set) data; III—missing values removed from most significant attributes; and IV—missing data imputed using KNN method.

Each algorithm group is tested internally in meta-algorithm using WEKA built in paired *t*-test statistical significance test tool. The selected confidence level is 95%. Actual significance numbers in results are not represented and not necessary in this intermediate comparison process. Only the indication if algorithm output result is significant (*p* ≤ 0.05) or not comparing to when base algorithm in group is used. If a particular algorithm appears to be significant among others, it is selected as the best performing algorithm; otherwise, the algorithm with the highest AUC is used. Similarly, if more algorithms appear to be significant, then one with the highest AUC value is used.

### 3.1. Removing Missing Values and Selecting Best Performing Classifiers for Scenario I

In order to get data without missing values, the suggested method has been applied as seen in Figure 1. This approach gave a significant increase of data records compared to the simple removal of instances with missing data. The increase varies from 26% to 64%, depending on the algorithm and optimal attribute set selected using the wrapper method (Table 3).

**Figure 3:**
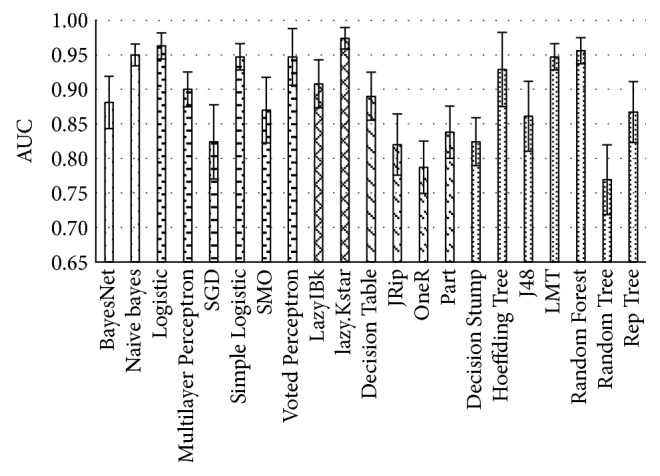
Averaged performance of classification algorithms on data without missing values with standard error bars.

Each algorithm performance output is represented as mean AUC, with standard error bars, of an averaged 10-fold cross-validation (Figure 3). Selected algorithms of each group are visible in Table 3.

When selected algorithms are combined into a final result, the joined list consists of following 9 attributes: *ALT*, *AST*, *albumin*, *pararenal shunt*, *liver stiffness*, *spleen stiffness*, *umbilical shunt*, *hepatic venous blood flow type*, and *splenic vein width*.

### 3.2. Classifying Original Dataset—Scenario II

**Figure 4:**
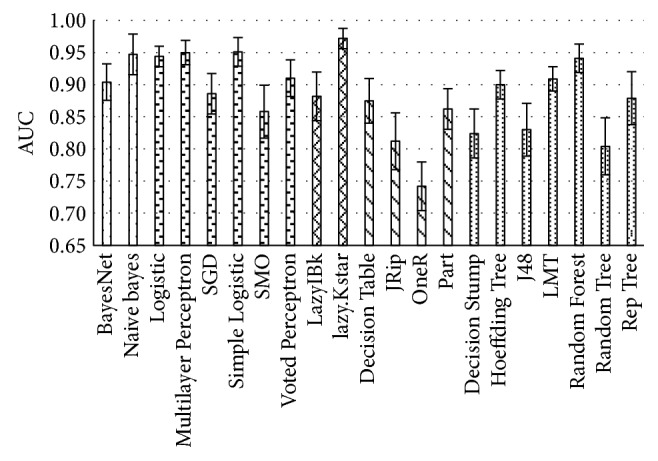
Performance of classification algorithms on data with unprocessed missing values.

In scenario II, classification algorithms have been tested with an unprocessed dataset having no missing values. Test results are visible in Figure 4, where mean AUC and standard error bars are plotted.

The five best performing algorithms have been selected in this case: Naïve Bayes, Simple Logistic, lazy.KStar, Decision Table, and Random Forest. Since the original data has some missing values scattered, there are more attributes for each algorithm generated. When selected algorithms are combined into a final result, the joined list consists of 16 attributes: *weight*, *pararenal shunt*, *hepatic venous blood flow type*, *haptic vein damping index*, *liver stiffness*, *spleen stiffness*, *portal vein width*, *prothrombin time*, *spleen length*, *spleen width*, *ALT*, *umbilical shunt*, *gender*, *PLT*, *albumin*, and *age*.

### 3.3. Removing Missing Data from Most Significant Attributes—Scenario III

In the third scenario, there were two highest rank attributes selected using a relief filter. A threshold of 0.1 has been selected to distinguish significant attributes from which to remove missing values. This way, any important attributes are fully filled with data while lower rank attributes are left untouched.

**Figure 5:**
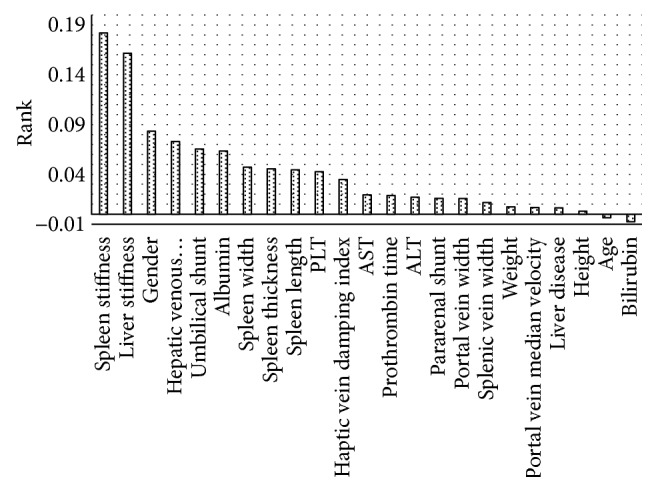
Ranked attributes using relief filter.

We can see in Figure 5 that two attributes stand out among others: spleen stiffness, having a rank of 0.18, and liver stiffness, ranked 0.16. Other attributes stay below the 0.1 threshold. After removing missing values from the highest ranked attributes, 99 records are left in the dataset.

**Figure 6:**
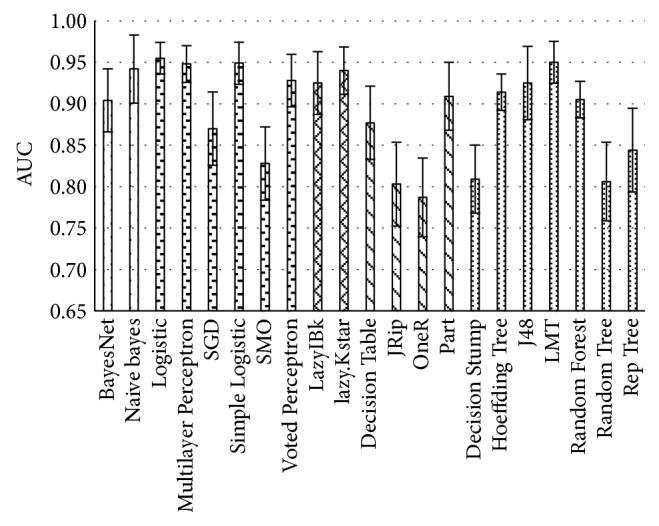
Performance of classification algorithms on data with missing values removed from top-ranked attributes.

Similarly, all five groups of algorithms were tested with the dataset, and the following best performing classifiers have been selected (Figure 6): Naïve Bayes, Logistic, lazy.Kstar, Part, and LMT. When the five best performing classification algorithms results are combined, the joined list consists of 16 optimal attributes: *gender*, *height*, *age*, *ALT*, *bilirubin*, *albumin*, *AST*, *prothrombin time*, *spleen width*, *pararenal shunt*, *portal vein median velocity*, *splenic vein width*, *hepatic venous blood flow type*, *haptic vein damping index*, *liver stiffness*, and *spleen stiffness*.

### 3.4. Imputing Missing Values Using KNN Method—Scenario IV

In this case, missing values were imputed using KNN method with Euclidian distance function. All 107 data records were preserved and all missing values were filled with nearest neighbor values.

**Figure 7:**
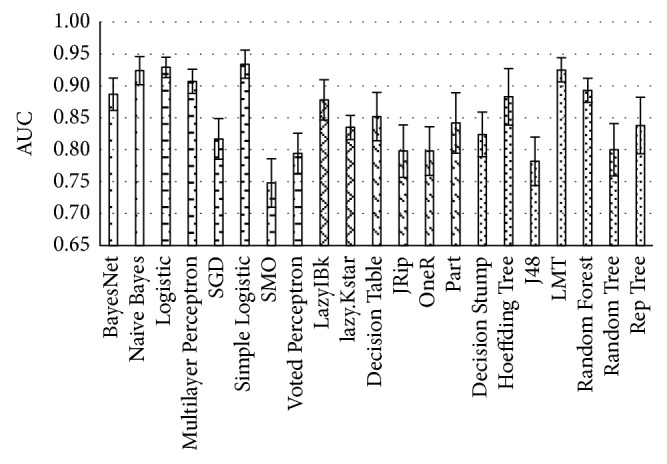
Performance of classification algorithms on data with missing values imputed using KNN method.

The best five algorithms were as follows (Figure 7): Naïve Bayes, Simple Logistic, lazy.IBk, Decision Table, and LMT tree. The joined list consists of 15 optimal attributes: *age*, *height*, *prothrombin time*, *spleen thickness*, *pararenal shunt*, *portal vein median velocity*, *haptic vein damping index*, *liver stiffness*, *liver disease*, *AST*, *albumin*, *spleen length*, *umbilical shunt*, *splenic vein width*, *gender*, and *spleen stiffness*.

In Table 4, we can see the numerical results of all four test scenarios. In each scenario, five algorithm groups are tested separately where statistical significance *t*-tests with selected significance level of *p* < 0.05 are performed. In each algorithm group, one algorithm is selected as “base” which is used for comparing other algorithms' significance. “0” means no significance; “1” means algorithm is significantly better against base; and “−1” means algorithm is significantly worse against base.

### 3.5. Meta-Algorithm Classification Results

**Figure 8:**
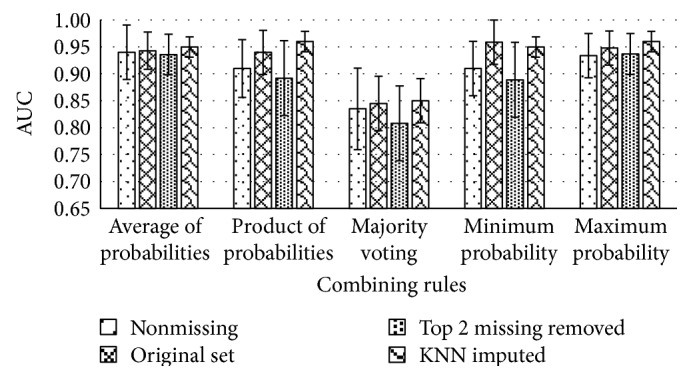
Mean AUC and standard error bars of all four scenarios and combining rules of meta-algorithm.

After the best performing algorithms and optimal attributes were selected, the last stage of the meta-algorithm combined the results for each best classifier into a single prediction.  Figure 8 represents a 10-fold cross-validation, where the mean AUC and standard error of all four scenarios are represented side by side.

We can see that classifying the dataset with KNN-imputed missing values gives the best results: AUC = 0.96 with a least standard error of 0.02. The maximum probability combining rule seems to be working best in this case. The *t*-paired significance test did not show the significance at confidence level of 95% when comparing results. In this case, the highest AUC result as winning was selected. For an unprocessed dataset, the results were slightly lower: AUC = 0.96 and standard error 0.04. The other two scenarios gave slightly lower results with AUC = 0.94 (Table 5).

We need to point out that in each scenario, a different number of records was used due to coping with missing values. Other factors may have also influenced each scenario's performance, such as different best classifiers selected inside the meta-algorithm, the data profile on each scenario, and varying sets of attributes.

## 4. Discussion

In this study, there has been an attempt to find an objective classification model that would replace the invasive PHT evaluation. The study shows that different classification algorithms produce differing classification results. Each algorithm brings its own benefits and flaws. Using a single classification algorithm may increase chances to overfit the dataset, which is quite limited having only 107 patient records. Therefore, an integration of the best algorithms into a meta-algorithm appears reasonable. Since real-world data always has missing values, four classification scenarios of data preprocessing have been tested with the meta-algorithm.

The first scenario included classification of data with nonmissing values. The number of data records has been maximized by using a proposed attribute selection and mapping method, which gave the maximum number of data records with the selected suboptimal attribute set. Instead of simply removing records with missing values, up to 28% of records were saved; however, the meta-algorithm did not yield the best classification result.

In the second scenario, better results were achieved with the original dataset. In this case, the algorithm produced higher accuracy and AUC. Since there are missing values in the dataset, classifiers are using more attributes to uncover deeper links within the data. The meta-algorithm required 16 attributes out of a total of 24. In addition, the number of records and optimal classifiers differs from that of the first scenario.

In the third scenario, all attributes were ranked by their importance using the attribute relief filter. Missing values were then removed from the most important attributes; however, the meta-algorithm gave slightly worse results than the second scenario where original dataset was used.

In fourth scenario, missing values were imputed using KNN method. Missing values were filled with the nearest neighbor value measured by Euclidian distance. This way, all 107 records were used in the dataset.

The meta-algorithm running on a dataset with missing values imputed using KNN method performs best. The highest mean AUC value of 0.96 and lowest standard error of 0.02 was achieved when using the maximum probability combining rule. Out of 24 attributes, 15 were selected as best performing using the threshold value of 1% for search. The set (*age*, *height*, *prothrombin time*, *spleen thickness*, *pararenal shunt*, *portal vein median velocity*, *haptic vein damping index*, *liver stiffness*, *liver disease*, *AST*, *albumin*, *spleen length*, *umbilical shunt*, *splenic vein width*, *gender*, and *spleen stiffness*) is required to run the meta-algorithm containing five classification algorithms: Naïve Bayes, Simple Logistic, lazy.IBk, Decision Table, and LMT tree. Selected attributes are not listed in order of importance. The algorithm-dependent wrapper algorithm is not capable of ranking them by importance. In order to find most related attributes to PHT, additional possible methods may be required.

We found several research projects that tried to classify patients according to HVPG using noninvasive methods. The comparison of the best meta-algorithm results with other research projects done in a similar field is visible in Table 6.

Not all research projects calculated AUC value, so we have included other metrics available, such as sensitivity and specificity combined into an F1 score, AUC, and accuracy. When comparing AUC values, our meta-algorithm gives the best results with a value of 0.96, which is close to the statistical liver TE-based method [14] with an AUC of 0.95, which was calculated from the same data we used. Despite a slight increase of performance, the meta-algorithm, with a number of different classifiers included, tends to be most robust and objective than any of the above.

From a clinical perspective, comparing the meta-algorithm to the second best research project where the liver TE attribute is used, it has some drawbacks. The number of required data fields is quite large to be used in an everyday clinical setting and some attributes are not usually used in a routine practice (e.g., the hepatic venous damping index), or if they are used, there is big chance that not all are performed on every patient with suspected PHT. In this context, the best performing “lazy.Kstar” algorithm with an AUC value of 0.97 (compared to 0.95 for liver TE alone) and the reduction in the number of attributes to five could be more acceptable (with regard to affordability and clinical practice). In order to get better objective results, more data is required for validation and fine-tuning parameters, so it is suggested that a flexible meta-algorithm could be a candidate for getting even better results with possibly less attributes required. In addition, the meta-algorithm could be used with the exclusion of data not used in everyday clinical practice.

## 5. Limitations and Further Research

There may be several influences in the objectivity of results and comparisons. First of all, datasets in each case varied in size because of removing records with missing values. The clinical dataset having 107 records could be too small for some classifiers to avoid specific errors. The meta-algorithm needs further testing and validating with a larger database. A larger variety of classification algorithms and their modifications can be included into a meta-algorithm in order to cover more possibilities. We have initially tried to change parameters of each algorithm to see if they give a significant change. The initial study showed that default parameter settings in WEKA work well, so no particular attention to parameters and settings were paid; however, this could be worth trying to optimize parameters in order to fine tune classification algorithms for even better results. In addition, the influence of missing values and their distribution need more investigating. Different methods of imputation of missing values need to be tested in order to find the best performing on a given data type. Along with the wrapper method of selecting an optimal set of attributes, there could be cost-based classification algorithms implemented in order to find consensus between objectivity and approved every day clinical practice. WEKA built in basic statistical test tool is sufficient for comparison of intermediate results inside meta-algorithm. For more in depth statistical tests, the other tools and methods such as analysis of variance (ANOVA) may be used.

## Figures and Tables

**Table 1:** Contents of data collected during clinical examination.

Demographical data	Age, gender
Clinical data	Height, weight, cause of chronic liver disease
Laboratory data	PLT, bilirubin, albumin, prothrombin time, ALT, AST
Instrumental data	Spleen width, spleen thickness, spleen length, HVPG
TE data	Liver stiffness, spleen stiffness
Blood stream data	Presence of umbilical or pararenal shunt, portal vein width, portal blood flow mean peak velocity, portal blood flow mean velocity, portal blood flow type (hepatopetal versus hepatofugal), hepatic vein blood flow type (triphasic, biphasic, or monophasic), hepatic vein damping index, splenic vein width

**Table 2:** Algorithms used in research.

BayesNet, Naive Bayes
Logistic, Multilayer Perceptron, SGD, Simple Logistic, SMO, Voted Perceptron
LazyIBk, lazy.Kstar
DecisionTable, JRip, OneR, Part
Decision Stump, Hoeffding Tree, J48, LMT, Random Forest, Random Tree, RepTree

**Table 3:** Best performing classification algorithms and attributes from five different groups.

Algorithm	Records	Increase	AUC	Best performing attributes
Naïve Bayes	91	40%	0.95	ALT, albumin, pararenal shunt, liver stiffness, spleen stiffness
Logistic regression	98	50.77%	0.96	Albumin, pararenal shunt, liver stiffness
lazy.Kstar	82	26.15%	0.97	Umbilical shunt, pararenal shunt, hepatic venous blood flow type, splenic vein width, liver stiffness
Decision Table	103	58.46%	0.89	Albumin, liver stiffness
Random Forest	107	64.62%	0.96	Age, albumin, pararenal shunt, liver stiffness, spleen stiffness

**Table 4:** The results of all four algorithms test scenarios.

	Scenario I			Scenario II			Scenario III			Scenario IV		
Algorithm	AUC	STDERR	*t*-test	AUC	STDERR	*t*-test	AUC	STDERR	*t*-test	AUC	STDERR	*t*-test
Bayes Net	0.88	0.04	Base	0.90	0.03	Base	0.90	0.04	Base	0.89	0.03	Base
Naive Bayes	0.95	0.02	0	0.95	0.03	0	0.94	0.04	0	0.92	0.02	0
Logistic	0.96	0.02	Base	0.94	0.02	Base	0.96	0.02	Base	0.93	0.02	Base
Multilayer Perceptron	0.90	0.03	0	0.95	0.02	0	0.95	0.02	0	0.91	0.02	0
SGD	0.82	0.05	0	0.89	0.03	−1	0.87	0.04	−1	0.82	0.03	−1
Simple Logistic	0.95	0.02	0	0.95	0.02	0	0.95	0.03	0	0.93	0.02	0
SMO	0.87	0.05	0	0.86	0.04	−1	0.83	0.04	−1	0.75	0.04	−1
Voted Perceptron	0.95	0.04	0	0.91	0.03	0	0.93	0.03	0	0.79	0.03	0
LazyIBk	0.91	0.03	Base	0.88	0.04	Base	0.93	0.04	Base	0.88	0.03	Base
lazy.Kstar	0.97	0.02	1	0.97	0.02	1	0.94	0.03	0	0.84	0.02	0
Decision Table	0.89	0.03	Base	0.88	0.03	Base	0.88	0.04	Base	0.85	0.04	Base
JRip	0.82	0.04	0	0.81	0.04	0	0.80	0.05	0	0.80	0.04	0
OneR	0.79	0.04	0	0.74	0.04	0	0.79	0.05	−1	0.80	0.04	0
Part	0.84	0.04	0	0.86	0.03	0	0.91	0.04	0	0.84	0.05	0
Decision Stump	0.82	0.03	Base	0.82	0.04	Base	0.81	0.04	Base	0.82	0.03	Base
Hoeffding Tree	0.93	0.05	1	0.90	0.02	0	0.91	0.02	0	0.88	0.04	0
J48	0.86	0.05	0	0.83	0.04	0	0.93	0.04	0	0.78	0.04	0
LMT	0.95	0.02	1	0.91	0.02	1	0.95	0.03	1	0.93	0.02	1
Random Forest	0.96	0.02	1	0.94	0.02	1	0.91	0.02	1	0.89	0.02	1
Random Tree	0.77	0.05	0	0.80	0.04	0	0.81	0.05	0	0.80	0.04	0
Rep Tree	0.87	0.04	0	0.88	0.04	0	0.84	0.05	0	0.84	0.04	0

**Table 5:** Comparison of best meta-algorithm results from each scenario.

Scenario	Combining rule	Acc, %	Sn	Sp	Attributes	AUC	Standard error
I	Avg of probabilities	88.46	0.75	0.92	9	0.94	0.05
II	Min probability	89.72	0.83	0.92	16	0.96	0.04
III	Max probability	86.87	0.79	0.89	16	0.94	0.04
IV	Max probability	88.92	0.80	0.81	15	0.96	0.02

**Table 6:** Comparison of classification results.

Attributes	Acc, %	Sn	Sp	F1	AUC	Classifier
Demographic, laboratory data, liver/spleen TE. Our method	89.72	0.83	0.92	0.87	0.96	Meta voting
Measuring hyaluronan and laminin in serum [17]	—	0.83	0.82	0.83	—	Logistic regression
Albumin, INR, ALT [32]	77.00	0.93	0.37	0.53	—	Predictive model
Demographic, laboratory data [19]	—	0.82	0.76	0.79	0.82	Regression
Serum, liver TE [18]	80.00	0.82	0.83	0.82	0.84	Neural network
Liver TE [14]	88.70	0.88	0.88	0.88	0.95	Statistical
